# Rapid Development of Improved Data-Dependent Acquisition Strategies

**DOI:** 10.1021/acs.analchem.0c03895

**Published:** 2021-03-31

**Authors:** Vinny Davies, Joe Wandy, Stefan Weidt, Justin J. J. van der Hooft, Alice Miller, Rónán Daly, Simon Rogers

**Affiliations:** † School of Computing Science, University of Glasgow, Glasgow G12 8QQ, United Kingdom; ‡ Glasgow Polyomics, University of Glasgow, Glasgow G12 8QQ, United Kingdom; § Bioinformatics Group, Department of Plant Sciences, Wageningen University, 6780 PB Wageningen, The Netherlands

## Abstract

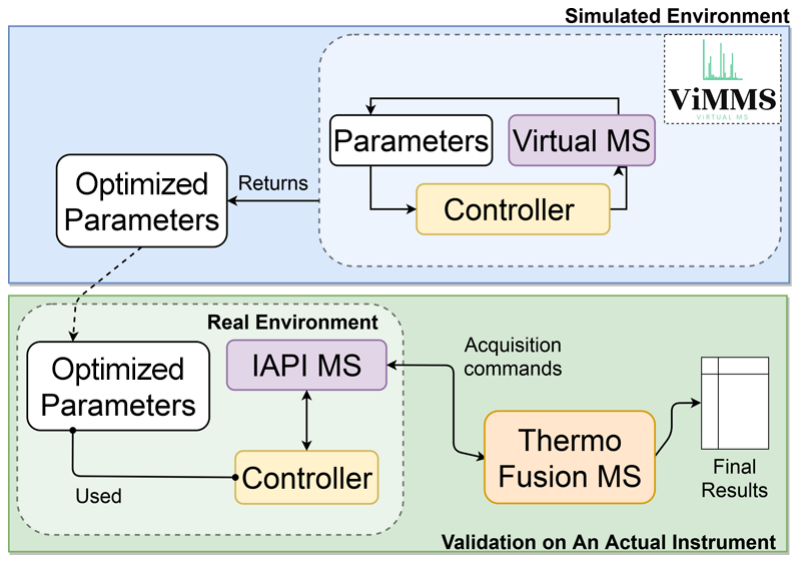

Tandem mass spectrometry (LC-MS/MS) is widely used to identify unknown ions in untargeted metabolomics. Data-dependent acquisition (DDA) chooses which ions to fragment based upon intensities observed in MS1 survey scans and typically only fragments a small subset of the ions present. Despite this inefficiency, relatively little work has addressed the development of new DDA methods, partly due to the high overhead associated with running the many extracts necessary to optimize approaches in busy MS facilities. In this work, we first provide theoretical results that show how much improvement is possible over current DDA strategies. We then describe an in silico framework for fast and cost-efficient development of new DDA strategies using a previously developed virtual metabolomics mass spectrometer (ViMMS). Additional functionality is added to ViMMS to allow methods to be used both in simulation and on real samples via an Instrument Application Programming Interface (IAPI). We demonstrate this framework through the development and optimization of two new DDA methods that introduce new advanced ion prioritization strategies. Upon application of these developed methods to two complex metabolite mixtures, our results show that they are able to fragment more unique ions than standard DDA strategies.

## Introduction

Tandem mass spectrometry (LC-MS/MS) is increasingly used in untargeted metabolomics to aid in the annotation of unknown chemical ions. Measured fragment (MS2) spectra for unknown ions can be used to aid annotation by direct comparison against spectral databases, machine-learning assisted comparison with structural databases (e.g., SIRIUS4^1^ and CFM-ID^2^), or analysis with metabolome data-mining tools such as molecular networking^3^ and MS2LDA substructure discovery.^4^

Crucial to all of these approaches is the acquisition of MS2 data. A good MS2 acquisition strategy ought to produce spectra of a high quality for as many of the ions present in the sample as possible. There are two main approaches that are used for MS2 acquisition in metabolomics: data-dependent acquisition (DDA) and data-independent acquisition (DIA). Recently work has been done to compare the two, but the results are inconclusive.^5^

DDA selects particular ions observed in MS1 survey scans for fragmentation and is used widely in metabolomics. In a typical DDA scheme, the set of *N* ions to fragment is determined based upon the most intense ions observed in the latest MS1 survey scan. Optionally, a dynamic exclusion window (DEW) can be included that avoids fragmenting the same mass-to-charge ratio (*m*/*z*) multiple times in succession, increasing the chance of fragmenting less-abundant ions. The chosen ions are isolated and fragmented by the MS in a series of MS2 scans, which are followed by the next MS1 survey scan, such that the duty cycle consists of one MS1 scan followed by up to *N* MS2 scans. A benefit of DDA is that the MS2 spectra emerge from the MS ready to use, i.e., each spectrum has been generated by fragmenting a small *m*/*z* isolation window (typically of the order of 1 Da) and will therefore normally contain fragments for a single chemical species. The disadvantages of DDA are the limited number of ions that can be fragmented within a single injection and the stochastic nature of fragmentation. Due to small variations in scan times, measured ion intensities can vary between runs, meaning ions with similar intensities can be prioritized differently. As such, if the same injection is run twice, different ions may be fragmented. It is also possible in some circumstances that multiple species can exist within one of the small isolation windows, typically resulting in chimeric spectra.^6^

DIA operates in a less-selective manner. Here, an MS1 scan is followed by one or more MS2 scans that do not depend on the MS1 scan. Each MS2 scan isolates a broader *m*/*z* range and can fragment many chemical species simultaneously. In theory, this means that all species in the data are fragmented and can be reanalyzed later if a new species becomes of interest. However, it is not necessarily guaranteed that spectra generated will be of sufficient quality to identify, especially in low-intensity species, although this is also the case with DDA. The resulting data require substantial processing to produce spectra assumed to come from a single chemical ion. This is done in software such as MSDIAL^7^ where (among other things) the chromatographic profile of precursor and product ions are matched. Spectra deconvolved in this way can then be used in the same manner as those produced by DDA.

There is no overall consensus as to which of these two schemes is best, and, where comparisons have been done, no clear conclusion is possible.^5^ Although the development of improved computational tools for spectral deconvolution has allowed more applications of DIA, DDA remains a popular choice due to the high spectral quality and the fact that little or no processing is required before the spectra can be used.

Given its popularity, surprisingly little work has been done to improve DDA performance for single injections in metabolomics. Some work has looked into DDA for multiple samples, specifically DsDA^8^ for multiple injections of different samples and AcquireX^9^ for repeated injections of the same sample, but these are not useful for single-injection DDA. Here, we address the problem of improving DDA coverage for a single injection, as a way of demonstrating how we can rapidly develop more general methods in silico.

One of the main criticisms of the performance of DDA (with respect to DIA) is its lower coverage: the proportion of ions that are fragmented. We start by computing the theoretically optimal performance for any particular injection, taking into account the uneven elution distribution of the ions. The results demonstrate that there is considerable room for improvement, motivating the development of better DDA strategies. Second, we describe how new strategies can be prototyped, implemented, optimized, and validated using a virtual metabolomics mass spectrometer (ViMMS),^10^ reducing the traditional need for a large amount of costly machine time. Recent novel additions to ViMMS mean that the same acquisition controllers can be used both in simulation and on real hardware. Finally, we describe two new DDA strategies prototyped in this way and demonstrate, through validation on two complex samples, their improvement over traditional DDA approaches.

## Methods

### Computing Theoretically Optimal DDA Performance

Computing the theoretically optimal DDA performance allows us to place an upper bound on the maximum number of fragmentation events that could occur, i.e., how many of the chemical ions present could a DDA method fragment at least once. This is not straightforward to compute as the limiting factor is often the coelution of too many ions in certain regions of the chromatogram.

To compute optimal performance, we start by defining the “true” set of chemical ions as the set of peaks picked from a TopN.mzML file by a commonly used peak-picking algorithm, such as those provided in MZmine2^11^ or XCMS.^12^ Picked peaks are represented by their bounding boxes (min and max retention time (RT) and *m*/*z* values). An MS scan schedule is created using the mean MS1 and MS2 scan times extracted from the TopN mzML file and a fixed value of *N* (the number of MS2 scans for each MS1 survey scan). This results in a list of scans and their respective scan start times. We create a bipartite graph where the two sets of nodes correspond to MS2 scans and peak bounding boxes from MS1, respectively. An edge, representing a potential fragmentation event, can be added between an MS2 scan and a bounding box if the MS2 scan time is within the RT limits of the bounding box, the MS1 scan preceding the MS2 scan also has an RT within the bounding box, and the peak’s intensity in this MS1 scan exceeds the minimum MS1 intensity for fragmentation.

Mirroring the standard acquisition process, we compute the optimal schedule by calculating a maximum matching for this graph using the Hopcroft–Karp algorithm (see Figure SI-3 in Supporting Information S4 for more details).^13,14^ A matching is a subset of edges within which no two edges share an end point. A maximum matching is a matching such that there is no other matching for the same graph that has more edges, meaning that we fragment the most peak-bounding boxes possible for the given graph. Computing the theoretically optimal maximum matching in this manner requires a knowledge of the entire run ahead of time, which will not be possible in practice. Nevertheless, it provides a useful upper bound on performance for evaluating new DDA schemes.

### Sample Preparation and Chromatography and MS Scan Settings

#### Sample Preparation

Two samples were used for our experiments to validate the performance of novel fragmentation strategies. Serum extract (QCA) was prepared from metabolite extraction of fetal bovine serum (South America origin (Gibco)) by dilution of 1/20 with water and addition of chloroform and methanol in a ratio of 1:1:3 (v/v/v). A beer sample (QCB) of Black Sheep Ale, 4.4%, was obtained. Sample extraction was performed by the addition of chloroform and methanol with a ratio 1:1:3 (v/v/v). A vortex mixer was used to mix the extracted solution. Centrifugation was performed to remove protein and other precipitates. Finally, the supernatant was removed, and the aliquot was stored at −80 °C.

#### Liquid Chromatography

Chromatographic separation with HILIC was performed for all samples using a Thermo Scientific UltiMate 3000 RSLC liquid chromatography system. A SeQuant ZIC-pHILIC column was used for a gradient elution with (A) 20 mM ammonium carbonate and (B) acetonitrile. We injected 10 μL of each sample into the column with initial conditions of 80% (B), maintaining a linear gradient from 80% to 20% (B) over 15 min, and finally a wash of 5% (B) for 2 min, before reequilibration at 80% (B) for 9 min. This used a constant flow rate of 300 μL/min and a constant column oven temperature of 40 °C.

#### Mass Spectrometry

A Thermo Orbitrap Fusion tribrid-series mass spectrometer was used to generate mass spectra data. Full-scan spectra were acquired in positive mode at a fixed resolution of 120 000 and a mass range of 70–1000 *m*/*z*. Fragmentation spectra were acquired using the orbitrap mass analyzer at a resolution of 7 500. Precursor ions were isolated using 0.7 *m*/*z* width and fragmented with a fixed higher-energy collisional dissociation (HCD) collision energy of 25%. The ACG was set as 200 000 for MS1 scans and 30 000 for MS2 scans.

## In Silico DDA Strategy Prototyping and Optimization

### Developing DDA Fragmentation Strategies

In our previous work, we introduced ViMMS,^10^ a simulator that could be used to evaluate different fragmentation strategies in silico. Fragmentation strategies are implemented as controllers in ViMMS. During simulation, controllers react to incoming scans and determine the next actions to perform by sending commands to the MS. Using the TopN controller as an example, the possible acquisition commands would be whether to perform a survey (MS1) scan or to generate fragmentation (MS2) scans.

Here we have extended ViMMS by creating a bridging code that allows controllers developed in ViMMS to be used directly on an actual MS. This bridge takes the form of a vendor-specific MS class in Python. Because of instrument availability, we currently support the Thermo Scientific Orbitrap Tribrid instruments through their Instrument Application Programming Interface (IAPI);^15^ however, the flexible design of our framework does not preclude supporting other vendors who offer real-time instrument control through an API.

**Figure 1:**
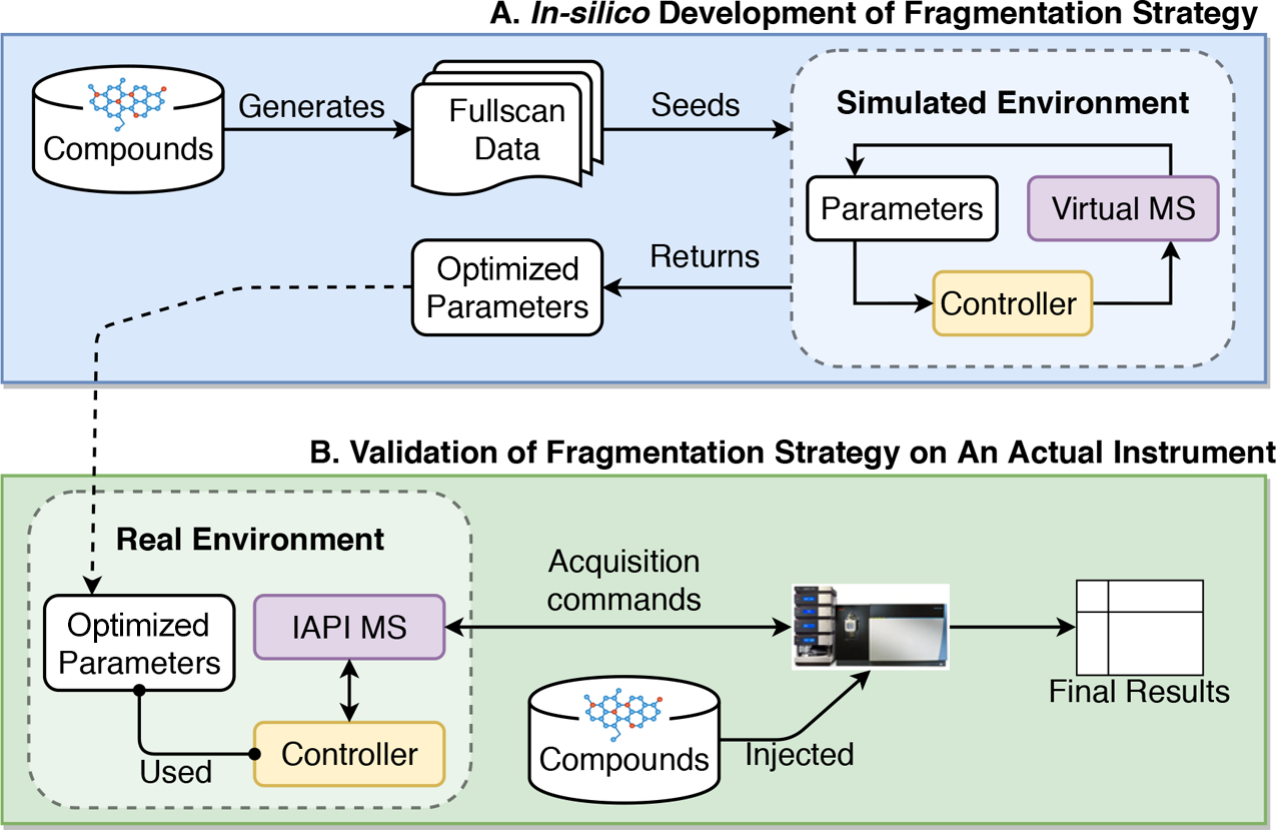
Flow diagram demonstrating the process of developing and optimizing a new fragmentation strategy. (A) Developing, testing, and optimizing the fragmentation strategy in silico. (B) Validating the developed fragmentation strategy using the simulated optimal parameters on the actual instrument.

Developing new methods in a simulation allows us to optimize them without having to rely upon costly MS time. Therefore, we propose the novel controller prototyping, optimizing, and validating pipeline shown in Figure 1. Full-scan (mzML) data is used to seed the virtual MS.^10^ The fragmentation controller under development is implemented in the ViMMS framework in the Python programming language. It runs in the simulated environment using the virtual MS. The performance of the controller is evaluated, and the best performing parameters are returned. For validation on the actual instrument, the optimized parameters from the simulation are used. The results from this validation experiment are reported as the final evaluation results. The same controller code (yellow boxes in Figure 1) works with both the simulated and the actual MS.

#### Performance Evaluation

We define two measures of performance to evaluate the effectiveness of different fragmentation strategies:

• *Coverage* is the number of picked peaks that contain a fragmentation event. In the absence of ground truth, we use peaks picked from full-scan data acquisition.

• *Efficiency* is defined as the ratio of the number of picked peaks that are fragmented to the number of MS2 scans, i.e., how many picked peaks are, on average, targeted by one MS2 scan. A perfect value of 1.0 indicates that each fragmentation event targets one unique picked peak.

To pick peaks we use mzMine2,^11^ with parameters provided in Supporting Information Table SI-1. Peaks are exported in the form of bounding boxes (*m*/*z* and RT min and max). To ensure that the results are not biased to one peak-picking algorithm, we also evaluated the methods using XCMS 3.6.1^12^ and Peakonly^16^ (see Supporting Information S3). MS2 fragmentation events are checked to see which peak bounding boxes they fall into (if any). The RT range of the bounding box is defined by the first and last MS1 scans that comprise the chromatographic peak.

#### Validation on Actual Instrument

For each serum (QCA) and beer (QCB) extract, we ran six injections: one full-scan (for evaluating coverage and efficiency), one TopN (using the controller optimized as part of the development of ViMMS^10^), and four injections for the new fragmentation strategies. To compute coverage and efficiency, peaks were picked from the mzML files for the full-scan data of the two samples. The IAPI bridge was used to let ViMMS controllers communicate with Thermo Orbitrap instruments, making it possible for the same controller codes to run unchanged in both the simulator and an actual mass spectrometer. Because of licensing constraints, we are unable to provide the source code of the IAPI bridge. Note that the ViMMS framework is designed to be easily ported between different instruments, including another Thermo instrument, or even other manufacturers such as, e.g., Waters, as long as a bridge could be developed to allow communications with the instrument in real time.

### SmartROI: A Flexible Fragmentation Strategy That Targets Regions of Interest in Real Time

#### SmartROI

Our first proposed new controller is motivated by the observation that a large number of MS2 scans in the TopN controller targeted ions that were not subsequently picked as peaks. The SmartROI controller keeps track of regions of interest (ROIs) in real time and only fragments peaks within ROIs. Creation of ROIs is the first step in many peak-picking methods, and therefore, fragmentation events outside ROIs are almost certainly wasted.^17^

SmartROI can be considered a variant on a TopN strategy in which the object being prioritized for fragmentation is the ROI instead of individual detected ions. As MS1 survey scans appear from the MS, and the set of ROIs is updated according to the ROI algorithm.^17^ ROIs that are not extended by the data from the MS1 scan are considered inactive and discarded. The remaining active ROIs are prioritized based upon intensity but only if they are available for fragmentation, determination of which is based on the following rules:

1. They must have an intensity in the most recent survey scan of greater than or equal to the minimum intensity for fragmentation.
2. If they have not been fragmented before, they are available.
3. If they have been fragmented before, then they are available if either of the following conditions are met:
  (a) Their intensity is higher by a factor α than when it was previously fragmented.
  (b) Their intensity has dropped by a factor β from its highest value since it was last fragmented.

Any ROI that does not meet these conditions is not available for fragmentation and will be ignored.

**Figure 2:**
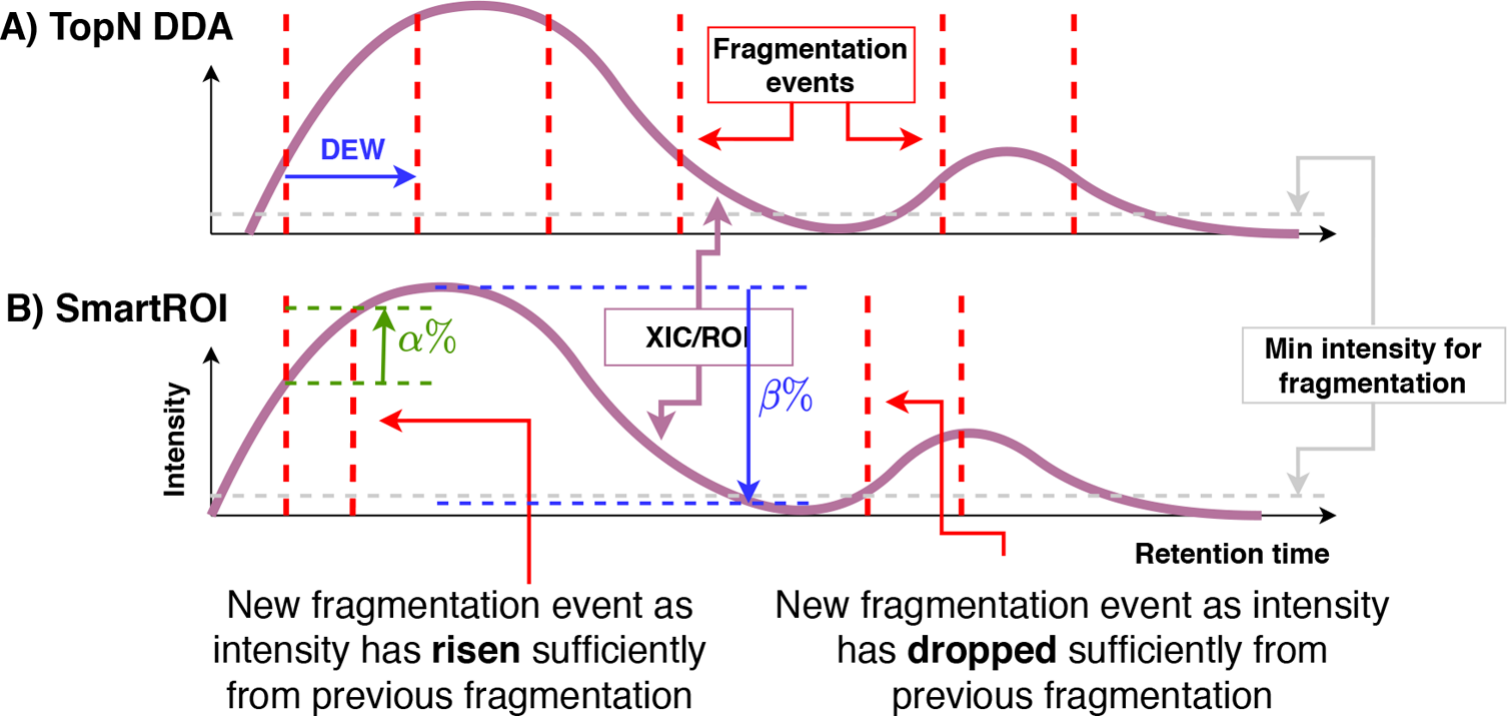
SmartROI compared with a TopN strategy. Keeping track of an ROI in real-time allows for better targeting of MS2 events.

This strategy can be seen in Figure 2. The upper plot shows a chromatogram (*x*-axis is retention time, and *y*-axis is intensity) and a possible set of fragmentation events using a standard TopN strategy. The dashed gray line shows the minimum intensity for fragmentation. Note that, in reality, fragmentation events would depend upon the other ions eluting at the same retention time, but it is easier to understand the approaches when considered in isolation. When the intensity falls below the minimum intensity, fragmentation ceases, starting again when it rises above the threshold. In the lower plot of Figure 2, the same chromatogram is shown for SmartROI. The first fragmentation event mirrors that in the TopN. The second is slightly earlier, being triggered when the intensity has increased by α%. This behavior is to ensure that we only fragment an ROI again if it has substantially increased in intensity. The SmartROI scheme then cannot fragment until the intensity has dropped by β% from the highest point since the previous fragmentation. However, the intensity is below the minimum intensity and so fragmentation does not occur until it has risen. The purpose of the β% drop is to ensure that we do not miss multiple peaks within the same ROI. The final fragmentation in the SmartROI example is triggered because the intensity has risen again by α%. SmartROI typically results in fewer, more precisely targeted fragmentation events than TopN.

#### Shifted SmartROI

In a standard duty cycle, we complete an MS1 scan, process it, and then perform up to *N* MS2 scans based on the result. Due to the additional complexity of updating the ROIs, calculating where to schedule the *N* MS2 scans takes longer in the SmartROI method (see Supporting Information Table SI-6). Therefore, there is a significant period where the MS stands idle between the MS1 and *N* MS2 scans. To overcoming this delay, we propose a slight variant to the controller. After the initial MS1 scan, we schedule *N* – 1 (or *N* – 2) MS2 scans, followed by an MS1 scan and 1 (or 2) MS2 scans related to the initial MS1 scan. While the final MS2 scans from from the initial MS1 scan are being completed on the MS, we process the second MS1 scan, again scheduling *N* – 1 (or *N* – 2) MS2 scans, followed by an MS1 scan and 1 (or 2) further MS2 scans. This means that we process each MS1 scan concurrently with other scans being completed on the MS, meaning that the machine sits idle for only a small amount of time, despite the complexity of the SmartROI method. The result here demonstrates how we could potentially fit a greater number of scans into one injection compared to the standard SmartROI method.

### WeightedDEW: A Fragmentation Strategy with Weighted Dynamic Exclusion Scheme

WeightedDEW generalizes the concept of the dynamic exclusion window. It is motivated by the problem of setting DEW width in standard TopN approaches: (i) too narrow and we waste MS2 scans repeatedly fragmenting the same ions, and (ii) too wide and we miss closely eluting peaks with similar *m*/*z*.

**Figure 3:**
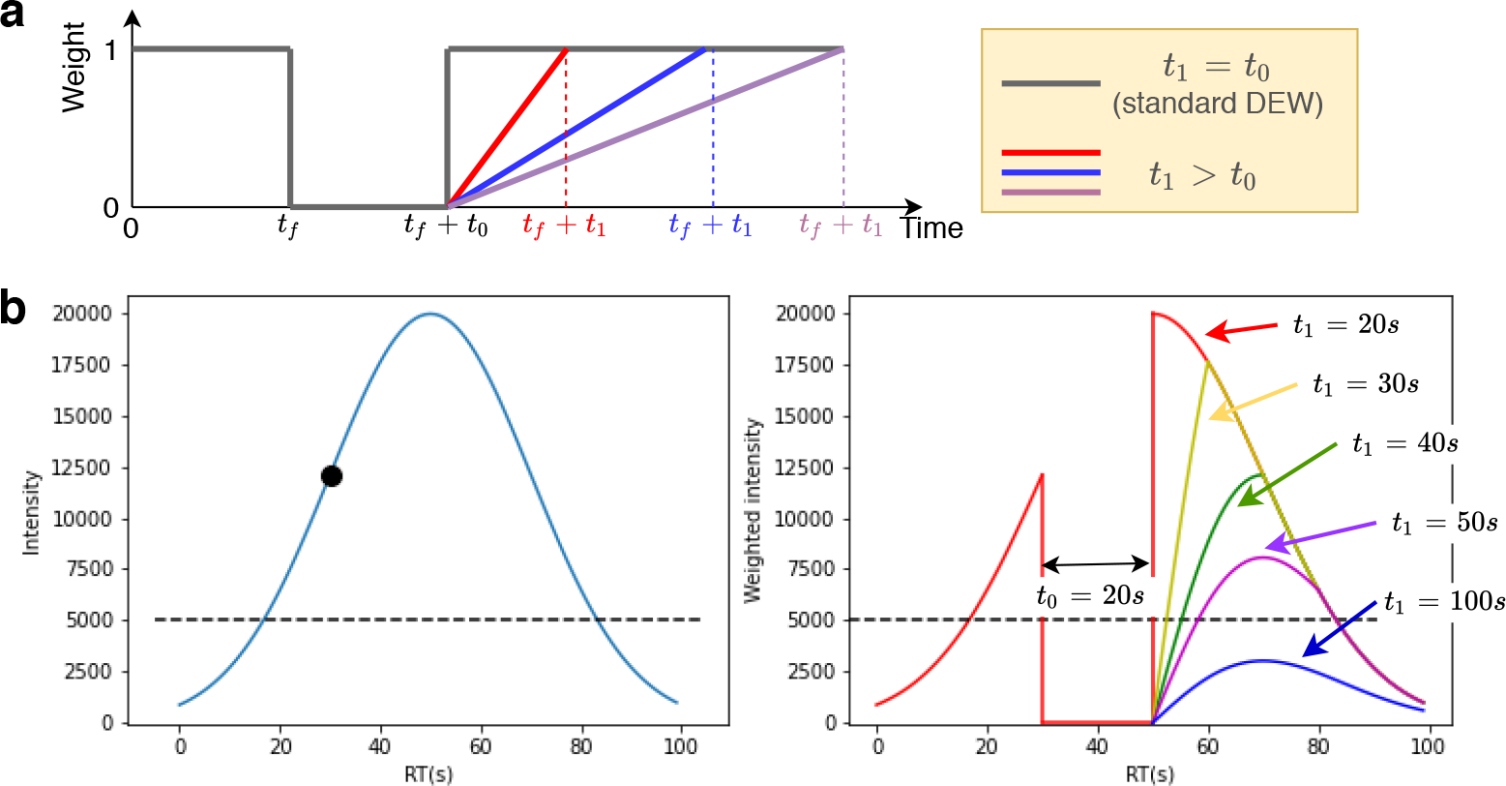
(a) Weight function in WeightedDEW. In standard DEW (*t*_1_ = *t*_0_), the weight is zero from the fragmentation event until *t*_0_ seconds has elapsed. In WeightedDEW, as *t*_1_ increases, the weight takes longer to return to 1. (b) Example chromatogram (left) showing a fragmentation event (black circle, 30 s) and minimum fragmentation intensity (dashed line). The weighted intensity (right) is zero until *t*_0_ (20 s) has elapsed. Different curves show the effect on the weighted intensity of increasing *t*_1_.

TopN DDA uses the intensity of the ion in the survey scan for fragmentation prioritization. When using a DEW, peaks are excluded from repeated fragmentation as long as their *m*/*z* and RT values are still within the dynamic exclusion window of previously fragmented ions. In a standard TopN DDA scheme, this can be thought of as prioritizing ions based upon the intensity multiplied by a binary indicator (which is 0 if the ion is still excluded by DEW and 1 otherwise). The result of multiplying the precursor ion intensities and the DEW indicator terms are then used to select the TopN ranked ions to fragment. WeightedDEW generalizes the binary DEW indicators to nonbinary weights. It is defined by two parameters: *t*_0_ and *t*_1_. The weight for a particular ion, *w*, observed at time *t* is given by
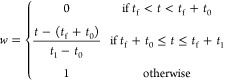
where *t*_f_ is the most recent time at which this *m*/*z* was fragmented. This function, for different values of *t*_1_, is shown in Figure 3a. A standard exclusion is applied for the first *t*_0_ seconds after fragmentation, after which the weight increases linearly from 0 at *t*_0_ to 1 at *t*_1_.

An example chromatogram and weighted intensity can be seen in Figure 3b, with a fragmentation at 30 s, *t*_0_ = 20 s, and *t*_1_ increasing from 20 to 100 s. WeightedDEW down-weights chromatograms for a period after their initial exclusion. Our hypothesis is that, by allowing for dynamic “exclusion” to be weighted linearly as a function of time and precursor ion intensity (rather than in a binary DEW manner), the system would be able to better prioritize smaller peaks that have not yet been fragmented.

## Results

### Optimal Results

The results of our optimal analysis show that, for both complex mixtures, the observed coverage from TopN DDA strategies are far from optimal, motivating the development of new methods. Optimal results were computed by picking peaks (see Supporting Information S4) from data acquired for the serum and beer extracts in full-scan mode. Scan timings were then taken from our TopN method, using the settings taken from the optimization presented in the validation of ViMMS.^10^ This forms two sets (the peaks and the MS2 scans) and can be formed into a bipartite graph matching problem. A maximum solution can then be found using a maximum matching algorithm,^14^ with the full results shown in Table SI-5. In summary, for both the serum and beer extracts, the coverage of the TopN method is significantly below the optimal: 656 (observed) vs 1542 (optimal peaks) and 1046 vs 2955 for serum and beer, respectively. Although we would never expect to be able to reach the optimum in practice (it requires global knowledge of the peaks and when they elute), the results demonstrate the considerable room for improvement available in DDA controller design.

### Controller Optimization

Both SmartROI and WeightedDEW were optimized using a grid search for coverage in simulation (more details are in Supporting Information S7). Supporting Information Figure SI-4 shows heatmaps of coverage for the serum and beer extracts for the SmartROI and WeightedDEW methods. For SmartROI, the parameter combinations α = 1000 and β = 0.1 performed well for both data sets and were chosen. For WeightedDEW, *t*_0_ = 15 s and *t*_1_ = 120 s were chosen. The grid search required 30 (SmartROI) and 36 (WeightedDEW) virtual injections for each of the serum and beer extracts, with each sample taking ∼1 h to produce in total. This is a significant time savings over running them on real equipment, demonstrating a clear advantage of optimizing in silico.

### Validation on Instrument

After parameter optimization, the controllers were validated on the real MS. We initially investigates the scanning frequency of the controllers by recording the time between scan start times in successive scans from the mzML file (Table SI-6). Here the timings for the MS1 scans represent both the time taken to do the MS1 scan and the time taken to process it and determine what scans to do next. ViMMS does allow the processing times for each controller to be tracked, but this was not implemented at the time of the injections. We expect the time taken to acquire scans on the instrument to be reasonably consistent, but for some controllers to be significantly slower at processing MS1 scans and prioritizing which scans to do next. For instance, due to the time needed to track ROIs in real time in the SmartROI controller, we expect SmartROI to have longer processing times than other controllers.

The results for the timings show that this is the case, with total processing and MS1 scan time taking 0.68 s for the SmartROI controller in the beer results, compared with 0.54 s for full-scan, 0.56 s for TopN, and 0.61 s for WeightedDEW. For both serum and beer extracts, the additional time between scans due to the processing is the equivalent of roughly one MS2 scan, motivating the development and evaluation of the shifted SmartROI controller, with shifts of 1 and 2 scans. WeightedDEW took slightly longer between scans than standard TopN. This is due to the fact that, while TopN can greedily move from the most intense MS1 peak down until it has scheduled *N* MS2 scans (or runs out of nonexcluded peaks), WeightedDEW has to compute the weights for all MS1 peaks above the minimum intensity threshold to ensure that it takes the topN weighted intensities into consideration. The time increase between TopN and WeightedDEW was not large enough to justify the use of a shifted controller for WeightedDEW.

Table 1 shows the performance in terms of coverage for the five controllers as well as the optimal performance as shown previously. In addition, we computed coverage based on peaks picked using XCMS and peakonly, both of which gave the same overall trends in performance with the new controllers outperforming the TopN controller (see Supporting Information S3). In both iterations of the serum and beer extracts, the best performing controller is the WeightedDEW. SmartROI performs the best with shifts of 2 and 1, respectively, for the beer and serum extracts, as the shift compensates for the extra processing time required. TopN is the worst performing method in both cases. The TopN comparison used was our own TopN controller and not the vendor TopN controller. This was due to the difficulty in comparing with the vendor controller due to the parallelization it employs. However, for context, we compared our new fragmentation strategies against a vendor TopN controller (with identical scan parameters), and our new controllers achieved higher coverage. A more detailed description of this comparison can be found in Supporting Information S2. Finally we also analyzed the intensities of common precursor ions fragmented by all methods and found that, while SmartROI and WeightedDEW slightly decrease precursor intensities at the time of fragmentation, this is compensated for by the increase in coverage of fragmented peaks that were missed by TopN (more details in Supporting Information S9).

**Table 1:** Coverage (Number of Picked Peaks Fragmented) for Each Controller for Both Iterations of the Beer and Serum Extracts, Where Peaks Have Been Picked Using MZmine2

	beer (4592 peaks)		serum (3032 peaks)	
method	iteration 1	iteration 2	iteration 1	iteration 2
TopN	1046		656	
WeightedDEW	1859	1768	1105	1226
SmartROI	1660	1546	991	1015
SmartROI (shift = 1)	1837	1740	1101	1193
SmartROI (shift = 2)	1838	1745	1040	1168
optimal (using TopN scan timings)	2955		1542	

We next consider the number of MS1 and MS2 scans produced by each method and the acquisition efficiency, shown in Table 2. We see a very wide range in the number of scans between the methods, explained predominantly by the variation in the number of MS2 scans. For the beer extract, where TopN and WeightedDEW typically create ∼6000 MS2 scans, the SmartROI controllers produce far fewer, resulting in a much higher efficiency. This is explained by the relative reluctance of the SmartROI controllers to refragment the same *m*/*z* values, even after a long time has elapsed. This increased efficiency allows more MS1 scans to be produced, which is useful if these files are also being used for peak picking and relative quantification. The more efficient controllers (e.g., SmartROI and WeightedDEW) perform better as the samples get more complex (Figure SI-5), where there would be more coelution of metabolites and, hence, more peaks to fragment at the same time.

**Table 2:** Total Number of Scans, Number of MS1 and MS2 Scans, and MS2 Efficiency (Eff) for the Two Experiment Iterations (Iter)^a^

		beer (4592 peaks)				serum (3032 peaks)			
Iter	method	total	MS1	MS2	Eff	total	MS1	MS2	Eff
	TopN	6404	583	5821	0.18	6317	575	5742	0.11
1	WeightedDEW	6282	572	5710	0.33	6235	567	5668	0.19
	SmartROI	4948	1050	3898	0.43	4271	1268	3003	0.33
	SmartROI (shift = 1)	5247	1056	4191	0.44	4299	1309	2990	0.37
	SmartROI (shift = 2)	5361	1054	4307	0.43	4353	1315	3038	0.34
2	WeightedDEW	6294	573	5721	0.31	6205	566	5639	0.22
	SmartROI	5078	1032	4046	0.38	4572	1237	3335	0.30
	SmartROI (shift = 1)	5395	1027	4368	0.40	4376	1329	3047	0.39
	SmartROI (shift = 2)	5413	1063	4350	0.40	4085	1414	2671	0.44

a Efficiency is the number of picked peaks that are fragmented divided by the number of MS2 scans.

## Discussion and Conclusions

In metabolomics experiments and studies, identifying spectra of interest is key to providing actionable scientific results. In standard experiments only a small number of the relevant species can be identified, as a result of there being no or poor quality spectra available for the species that the experiment has shown to be of scientific interest. Being able to acquire MS2 spectra for more species (increased coverage) improves the ability to annotate ions in an LC-MS/MS analysis and increases the chance of having spectra for the species of interest. Developing new acquisition methods that improve coverage is therefore a logical way to improve metabolomics experiments.

However, developing new acquisition methods has typically required extensive experimentation on the MS apparatus, which could be expensive and time-consuming. Here we demonstrated how new DDA strategies can be rapidly developed and prototyped in silico and then validated on the machine. Additionally we introduce a framework to support this development process by extending the capability of ViMMS^10^ so it could easily run fragmentation strategies implemented as controllers in the simulator on real MS equipment with minimal change to the code. A similar development process can be used for DIA, with new methods developed in ViMMS and the estimated spectra they produce using a deconvolution method such as MSDial^7^ compared against the known spectra put into the ViMMS framework.

Using this iterative design, prototype, and validation process, we presented two new DDA strategies that both considerably outperform a conventional TopN strategy that prioritizes ions for fragmentation based on intensity alone. In the first, SmartROI, we use an ROI detection algorithm commonly used for peak picking to only fragment molecules that are within real-time ROIs and are therefore likely to be picked as peaks. In the second, WeightedDEW, we generalize the dynamic exclusion window approach to a real-valued weighting scheme, allowing previously fragmented ions to smoothly rise up the priority list as their intensity remains high. In both cases, improved performance in silico was mapped to improved performance in reality, instilling confidence in the simulation procedures. Although the WeightedDEW controller outperformed the SmartROI in our chosen performance measure, we believe that both have utility. WeightedDEW is computationally straightforward, as demonstrated by its similar processing time to TopN, and it produces higher coverage compared to the alternatives here investigated. SmartROI requires more computational time but also offers more direct control in how often an ROI will be fragmented. The tracking of ROIs in real time also offers the advantage of further method development. For example, it should be possible to predict, in real time, if an ROI contains a peak or not and only fragment those predicted peaks. The increased efficiency of SmartROI also suggests that it would perform better in more crowded mixtures than those presented here. For example, background signals where the intensity values do not change much could potentially be fragmented multiple times in a standard TopN DDA scheme, but in SmartROI it will only be fragmented once. This is possible in SmartROI even without having a prior knowledge of which is the background ion; rather, it is accomplished through tracking of regions of interests in real time.

When optimizing our controllers, we chose to maximize the fragmentation coverage. MS2 scan parameters have remained constant throughout, so it is not the case that we have increased coverage at the expense of data quality, as would be the case if, for example, reduced scan resolutions were used. All of our MS2 scans were performed in the orbitrap mass analyzer to obtain high-resolution fragmentation data. It would be possible to improve coverage of all methods by performing MS2 analysis in the linear ion trap mass analyzer and fully make use of the possible parallelization.^18^ The optimization procedure proposed here is independent of any particular figure of merit: any other measure of MS2 acquisition quality could be used in place of coverage if considered more appropriate.

In addition, we have also shown how an optimal limit of DDA performance for a particular mixture can be computed via a bipartite graph matching scheme. This limit provides context for acquisition analysis results: for the two complex samples analyzed here, we are far from reaching these theoretical maxima, suggesting that much more optimization is possible. At the same time, this provides a framework for future DDA and DIA method optimization studies to perform benchmarking when applied to the samples used in their studies.

For validation on actual instruments, our proposed framework at the moment is limited to supporting the Thermo Fusion Tribrid instrument through the manufacturer’s provided IAPI. The modular nature of our software means that all controllers communicate with the instrument through bridging code, and therefore, the same controller implementations could easily run on different hardware if a real-time API is available from the manufacturers. For instance, Waters instruments could be supported by developing an appropriate bridge from our framework to communicate with the Waters Research Enabled Software (WREnS) API.

We conclude that there is much further improvement possible in the development of DDA strategies. We show how the use of a simulation system to optimize such strategies can rapidly lead to improvements. We demonstrate two such acquisition strategies, both exceeding performance over a TopN controller in terms of coverage (number of unique picked peaks that are fragmented). Finally, the flexibility of the framework allows future development of methods for multiple injections, in a similar manner to DsDA or AcquireX.

## Supporting Information Available

Peak-picking parameters, scan-timing analysis and comparison with vendor method, results for alternative peak-picking methods, computing theoretical fragmentation bounds, optimality results, scan timings for different controller methods, in silico optimization of controllers, and performance comparison for different data set sizes (PDF)
